# Antibacterial, antioxidant, and anticancer potential of green fabricated silver nanoparticles made from *Viburnum grandiflorum* leaf extract

**DOI:** 10.1186/s40529-024-00411-5

**Published:** 2024-01-22

**Authors:** Hina Talib, Ansar Mehmood, Muhammad Shoaib Amjad, Amna Mustafa, Muhammad Abdul Rauf Khan, Muhammad Raffi, Rizwan Taj Khan, Khawaja Shafique Ahmad, Huma Qureshi

**Affiliations:** 1 Department of Botany, University of Poonch Rawalakot, Rawalakot, Azad Kashmir 12350 Pakistan; 2 Department of Botany, Women University of Azad Jammu and Kashmir Bagh, Bagh, 12500 Pakistan; 3 Birmingham Institute of Forest Research, University of Birmingham, Birmingham, B15 2TT UK; 4 Department of Physics, University of Poonch Rawalakot, Rawalakot, Azad Kashmir 12350 Pakistan; 5 Department of Materials Engineering, National Institute of Lasers and Optronics (NILOP), Lehtrar Road, Nilore, Islamabad 45650 Pakistan; 6 Department of Botany, University of Azad Jammu & Kashmir, Muzaffarabad, Pakistan; 7 Department of Botany, University of Chakwal, Chakwal, 48800 Pakistan

**Keywords:** Green fabricated Ag nanoparticles, Physiochemical analysis, Antibiotics resistant bacteria, Rhabdomyosarcoma cancer cell lines, 2,2-diphenyl-1-picrylhydrazyl, MTT assay

## Abstract

**Background:**

Recently, researchers are focusing on creating new tools to combat the antibiotic resistant bacteria and malignancy issues, which pose significant threats to humanity. Biosynthesized silver nanoparticles (AgNPs) are thought to be a potential solution to these issues. The biosynthesis method, known for its environmentally friendly and cost-effective characteristics, can produce small-sized AgNPs with antimicrobial and anticancer properties. In this study, AgNPs were bio-fabricated from the distilled water and methanolic extracts of *Viburnum grandiflorum* leaves. Physio-chemical characterization of the bio-fabricated AgNPs was conducted using UV-visible spectroscopy, scanning electron microscopy, energy dispersive X-ray, and X-ray diffraction analysis.

**Results:**

AgNPs produced from the methanol extract were smaller in size (12.28 nm) compared to those from the aqueous extract (17.77 nm). The bioengineered AgNPs exhibited a circular shape with a crystalline nature. These biosynthesized AgNPs demonstrated excellent bactericidal activity against both gram-negative (*Pseudomonas aeruginosa*) and gram-positive (*Staphylococcus aureus*) bacteria. Highest antibacterial activity was observed with the methanol extract against *P. aeruginosa* (14.66 ± 0.74 mm). AgNPs from the methanol extract also displayed the highest antioxidant activity, with an IC_50_ value of 188.00 ± 2.67 μg/mL against 2,2-diphenyl-1-picrylhydrazyl (DPPH). Furthermore, AgNPs exhibited notable cytotoxic activity against Rhabdomyosarcoma cell line (RD cell) of human muscle cancer cell. The IC_50_ values calculated from the MTT assay were 26.28 ± 1.58 and 21.49 ± 1.44 μg/mL for AgNPs synthesized from aqueous and methanol extracts, respectively.

**Conclusion:**

The methanol extract of *V. grandiflorum* leaves demonstrates significant potential for synthesizing AgNPs with effective antibacterial, antioxidant, and anticancer actions, making them applicable in various biomedical applications.

## Background

For decades, nanotechnology has evolved as a pivotal field of study, offering distinctive properties and diverse applications across industries such as agriculture, food, textiles, electronics, and most significantly health care. It plays a crucial role in drug delivery, therapy, diagnosis, and biosensing, contributing significantly to humanity (Burdusel et al. 2018; Erci et al. 2018). Nanoparticles (NPs) have well-known uses in the medical fields due to their tiny size and remarkable properties, including high surface area, chemical, optical, magnetic, and mechanical characteristics (Tanase et al. 2019). Recently, AgNPs have received extensive research attention for their broad-spectrum actions (Kalishwaralal et al. 2009), serving as alternatives to other inorganic metallic NPs in various biomedical such as antimicrobial, antiviral, antibacterial, antioxidant, and anticancer and pharmaceutical industries (Nagarajan et al. 2019; Al-Shmgani et al. 2017; Deya and Bellotti 2017).

To meet the growing demand for commercially available nanoparticles, the extracellular production of inorganic NPs via a green synthesis method has become increasingly popular. This approach offers a simple, environment friendly, and single-step procedure, eliminating the need for complex equipment and hazardous chemicals (Karmous et al. 2020; Nadaroglu et al. 2017). The plant extracts, as reducing, capping, and stabilizing agents, not only reduce costs of maintaining microorganism culture but also provide a more stable NPs with desired shape and size (Thakur and Mohan 2019; Mousavi et al. 2018). Numerous studies have highlighted the importance and novelty of AgNPs synthesized from plant extracts (Ijaz et al. 2022; Kemala et al. 2022; Barabadi et al. 2021).

Plant extract-generated AgNPs, typically ranging from 1 to 100 nm, exhibit unique physicochemical properties, including high surface area, shape, size, electric conductivity, and optical activity. These properties make them effective antibacterial, antioxidant, and anticancer agents. The green synthesized AgNPs show toxicity towards bacteria and cause cell lysis. By attaching to DNA or denaturing ribosomes, they also stop ribosomes from synthesizing proteins and DNA from replicating (Schrofel et al. 2014). The bio-fabricated AgNPs demonstrated substantial cytotoxic activity towards HeLa cell lines, human epithelial cancer (Hep-2), and prostate cancer (PC-3) (Sukirtha et al. 2012; Jacob et al. 2012; He et al. 2016). They also serve as antimicrobials in surgical instruments, external anti-infection creams, and anti-cancer medicines (Sondi and Sondi 2004).

This study involves the leaf extracts of *Viburnum grandiflorum* Wall. exDC. for synthesizing AgNPs. It is a medicinal evergreen deciduous plant from the Viburnaceae family and is indigenous to the Himalayan area. This shrub is associated with a variety of biological functions, including traditional applications as a diuretic, antispasmodic, and sedative. The twigs are used as teeth brushes and liver defenders with anti-inflammatory effects (Yatoo et al. 2018). Despite reports on the antibacterial, antioxidant, and anticancer properties of AgNPs from plant extracts, no study has explored the production of AgNPs from *V. grandiflorum* leaves and their specific properties. Additionally, most studies use the plant’s aqueous extract, but we utilized both aqueous and methanol extracts to compare their outcomes. The primary goals of this study were to create and characterize AgNPs from *V. grandiflorum* extracts, assess their bactericidal activity, and evaluate their potential antioxidant and anticancer activities.

## Methods

### Plant material and extract preparation

Fresh *V. grandiflorum* leaves were harvested from healthy plants in Nakker, located in district Sudhnoti of Azad Kashmir, Pakistan. Plant identification was facilitated by consulting the flora of Pakistan, and the scientific name was confirmed through the Plant List (http://www.theplantlist.org). A voucher number was assigned to the plant specimen on herbarium sheet, which was then submitted to the Herbarium at the University of Poonch Rawalakot, Pakistan. The harvested leaves underwent a thorough cleaning with tap water to remove any residual dust and were subsequently air-dried in the shade at room temperature. After that, an electric grinder was used to finely grind the leaves. Ten grams of the resulting powder was mixed with 100 mL of distilled water in a 250 mL flask to prepare an aqueous extract, which was incubated for three days in a shaking incubator. Following that, Whatman No. 42 filter paper was used to further purify it. A same process was replicated for the methanol (Sigma-Aldrich, USA) extract. Both the aqueous and methanol extracts were employed in the bio-fabrication of AgNPs.

### Synthesis of AgNPs

For AgNPs synthesis, 20 mL of *V. grandiflorum* leaf extract was mixed with 80 mL of 1 mM silver nitrate (AgNO_3_, Sigma-Aldrich, USA). The reaction solution was incubated for 24 h at room temperature, and a change in color of the reaction solution was observed, as reported by Khalil et al. (2023). Subsequently, the reaction solution underwent centrifugation at 10,000 rpm for approximately 10 min. Following centrifugation, the AgNPs pellet was washed three times with distilled water and once with acetone. The resulting AgNPs were freeze dried to obtain them in powder form (Krishnaraj et al. 2012). The AgNPs produced from the aqueous extract and methanol extract are denoted as Aq-AgNPs and Met-AgNPs, respectively.

### UV–visible spectroscopy

UV-visible spectroscopy was employed to illustrate the reduction of Ag^+^ in the reaction solution. Pure water served as a reference, and a small quantity of colloidal solution was scanned within the wavelength range of 300–700 nm. UV-Visible spectroscopy analysis was conducted using a PerkinElmer Lambda 950 UV/Vis spectrometer (Oluwaniyi et al. 2016).

### Scanning electron microscopy (SEM)

SEM was utilized to unveil the morphological characteristics of AgNPs. After being suspended in double-distilled water for 10 min, the AgNPs powder underwent sonication. The resulting sample was then applied onto a carbon-coated copper grid and exposed to a mercury lamp for complete drying. Field emission scanning electron microscopy (MIRA 3 XM) was employed for the morphological analysis (Jyoti et al. 2016). Additionally, EDX coupled with SEM was applied to analyze the chemical composition of the AgNPs.

### X-ray diffraction (XRD) analysis

For XRD investigation, purified AgNPs were freeze-dried and then subjected to an XRD analysis using a diffractometer (Bruker D8 Diffractometer) with Cu K- radiation at a wavelength of 1.54 nm.

### Antibacterial activity

The bactericidal activity of plant extracts and AgNPs against clinically isolated bacteria, such as *Pseudomonas aeruginosa* and *Staphylococcus aureus*, was evaluated using the disc diffusion assay (Pal et al. 2007). Bacterial species were acquired from combined military hospital (CMH) Rawalakot. A nutritional medium was prepared by dispersing 14 g of Muller Hinton agar (Merck, USA) in 500 mL of distilled water. All glass components, disc (6 mm in diameter made from Whatman filter paper no. 1), and nutritional medium were autoclaved for 15 min at 121 °C. The sterilized nutrient agar medium and pure bacteria culture (1.5 × 10^8^ CFU/mL) were poured into petri dishes. Subsequently, filter paper discs permeated with AgNPs and plant extracts were strategically placed on the nutrient agar medium. The petri dishes were then incubated at 37 °C for 24 h. To quantify the antibacterial efficacy, the zone of inhibition surrounding the discs was measured in millimeters (mm).

### Antioxidant activity

The 2,2-diphenyl-1-picrylhydrazyl (DPPH) (Sigma-Aldrich, USA) method was used to assess the antioxidant activity (Bhakya et al. 2016). As a standard, ascorbic acid was used. AgNPs in various concentrations (125, 250, 500, and 1000 μg/mL) were added to the 1 mL DPPH solution and properly mixed. The solution was then kept at room temperature for 30 min. A UV-Vis spectrophotometer was used to measure the absorbance at 517 nm. Methanol and distilled water were employed as a blank solution, and DPPH (all components except the sample) was utilized as a reference. The antioxidant activity is calculated using the formula provided below (Khane et al. 2022).

$$ \% \,{\rm{antioxidant}}\,{\rm{activity}}\,{\rm{ = }}\frac{{pc - ps}}{{pc}} \times 100$$

‘ps’ stands for AgNPs/ascorbic acid absorption, while ‘pc’ stands for control absorbance. The regression line equation was used to calculate the IC_50_ for the investigated samples at various concentrations.

### Anticancer activity

The anticancer activity was confirmed following the procedure of Lyons et al. (1998) with small modifications. Rhabdomyosarcoma cell line (RD cell) of human muscle cancer cell obtained from the National Institute of Health (NIH), Islamabad. These cell lines were then created at Pakistan Institute of Engineering and Applied Sciences (PIEAS) Phyto medicine facility in Islamabad. In culture flasks at 37 °C and a CO_2_ stream in a CO_2_ incubator, RD cells were multiplicated. Eagle’s Minimal Essential Medium (EMEM) was applied to facilitate the cells beneficial replication. More than 80% of merged cells were utilized in a culture flask. The cells were taken out of the growing flask and cleaned in PBS that had been autoclaved. After adding 0.5 mL of trypsin, the culture flask was allowed to sit for two to three minutes. The cells were taken out of the incubator, frozen to separate any clusters, and examined under an inverted microscope. Cells were combined with 5 mL of EMEM medium using a pipette (10%). In a brand-new flask, 2.5 mL of single cell suspension was added. In 96-well plates with 200 μL of growth medium, 5 × 10^3^ cancer cells were seeded each well. The cells were subjected to generated AgNPs at increasing concentrations after 24 h. After 24 h, the media was swapped with an MTT solution (10 μL, 5 mg/mL/well) prepared in PBS, and the cells were cultured for an extra 3 hours in a humidified incubator at 37 °C with 5% CO_2_. A micro titer plate reader operating at 595 nm was used to measure absorbance after the plates were slightly stirred for 1 min with isopropanol (50 mL) poured to each well (Bio-Rad). The percentage of viable cells was calculated by using the following equation.

$$\% \,{\rm{cell}}\,{\rm{viability}}\,{\rm{ = }}\frac{{{\rm{Absorbance}}\,{\rm{of}}\,{\rm{AgNPs}}\,{\rm{treated}}\,{\rm{cells}}}}{{{\rm{Absorbance}}\,{\rm{of}}\,{\rm{untreated}}\,{\rm{cells}}}} \times {\rm{100}}$$

The means and standard error of means of the data were used to represent the outcomes of three independent tests.

### Data analysis

Each test was run three times. Analysis of variance (ANOVA) was used to evaluate the data, and the least significant difference (LSD) method was used to compare the means.

## Results and discussion

### Synthesis of silver nanoparticles (AgNPs)

**Fig. 1:**
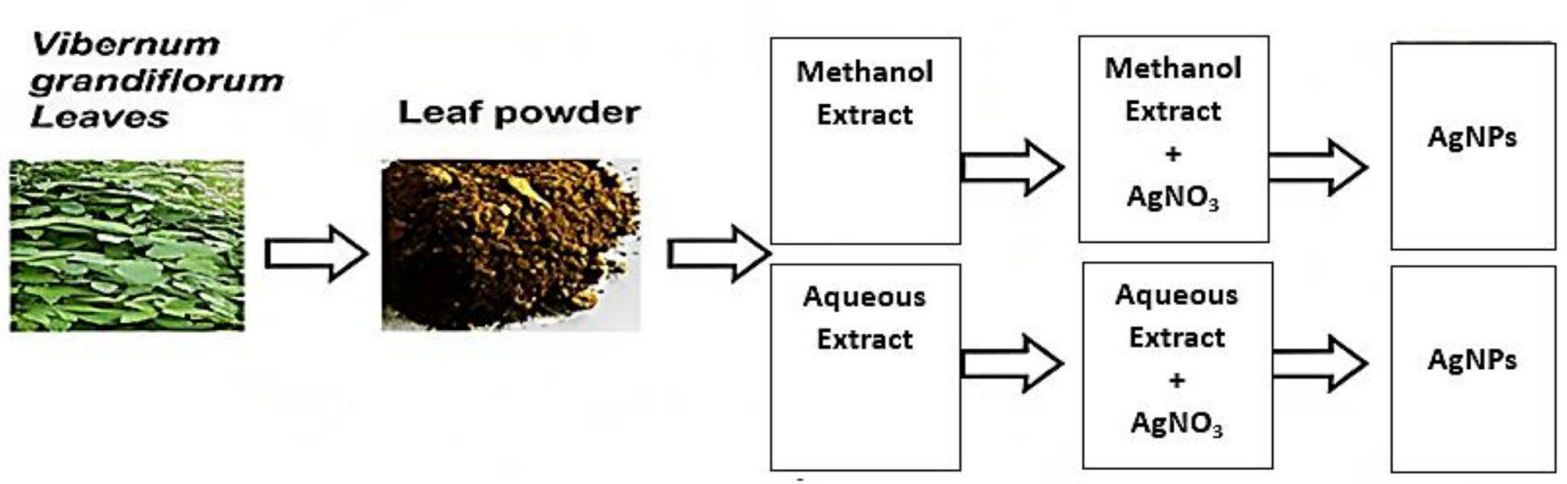
Schematic diagram for the synthesis of AgNPs

The synthesis of AgNPs was achieved by treating the plant extracts (both aqueous and methanol) with an AgNO_3_ solution. The color of the colloidal solution underwent noticeable changes, turning from greenish to brown in the case of the aqueous extract and from light to dark brown in the case of the methanol extract within 24 h of introducing a pure solution of 1 mM AgNO_3_ into the plant extracts. This alteration in color is a well-established phenomenon observed when silver ions are reduced to AgNPs, as reported by Alsalhi et al. (2016). Similar hue changes from yellowish to dark brown have been documented by Widatalla et al. (2022) and Ali et al. (2016). The color transformation is attributed to the conversion of Ag^+^ ions into Ag^0^ and the incidence of vibrations in the Surface plasmon resonance (SPR) as elucidated by Ahmed et al. (2019) and Baran et al. (2018). Singh et al. (2010) further suggested that electron oscillation and SPR play pivotal roles in color changes. The process for the green fabrication of AgNPs is shown in Fig. 1.

### UV-visible spectroscopy

**Fig. 2:**
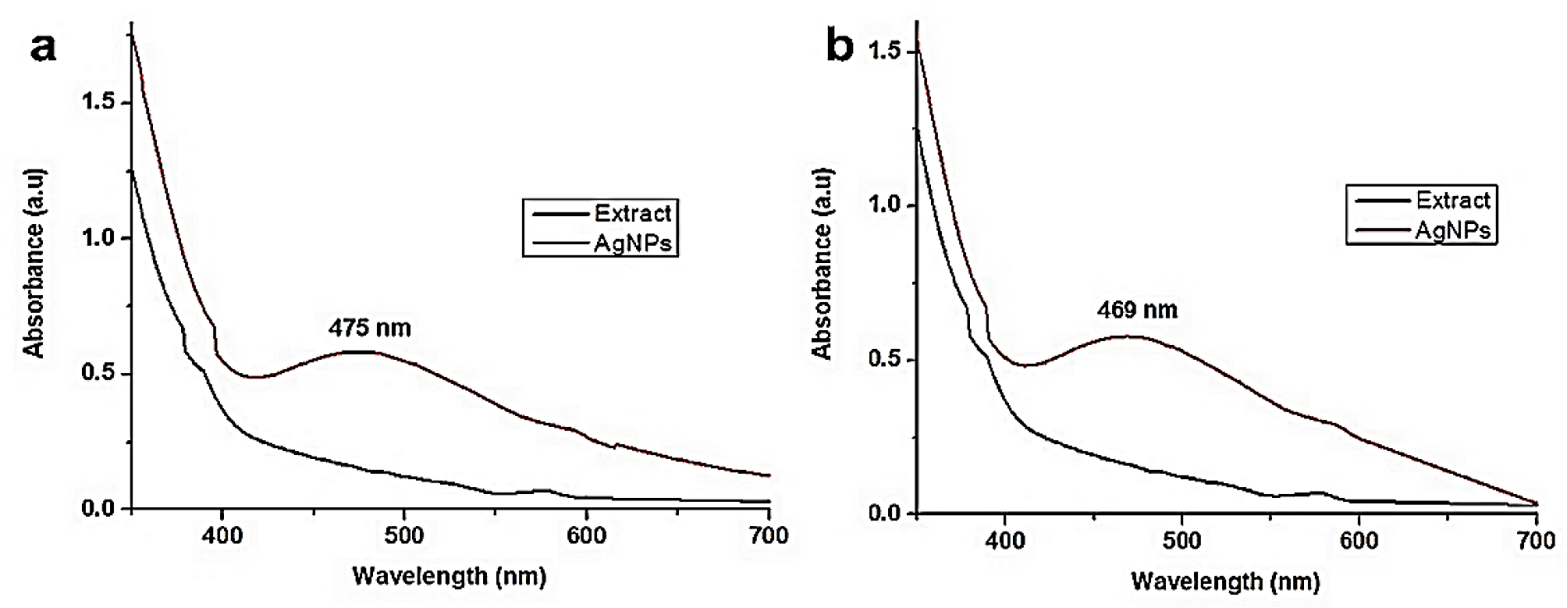
UV-visible spectra of AgNPs. AgNPs were synthesized from aqueous (**a**) and methanol (**b**) extracts of *Viburnum grandiflorum* leaves

The UV-Vis spectroscopy method was employed to investigate indirectly the bio-reduction of AgNPs generated from an aqueous solution of AgNO_3_. The choice of solvent for plant extraction was found to influence the reduction of Ag^+^ to Ag^0^. The UV-Vis spectrum of AgNPs produced from the aqueous extract is shown in Fig. 2a, revealing an absorption peak occurring at 475 nm. Conversely, the plant extract itself did not exhibit any absorption peak in this region. Figure 2b illustrates the UV-Vis spectrum of AgNPs synthesized from the methanol extract, with the absorption peak occurring at approximately 469 nm. It is well-established that AgNPs typically exhibit an absorption peak in the 400–500 nm range (Widatalla et al. 2022; Aziz et al. 2017; Mittal et al. 2012). Interestingly, we observed a shift in the absorption peak from 475 to 469 nm (a blue shift) when methanol extracts were utilized for AgNPs production in comparison with that of aqueous extracts. This shift is linked to alterations in the size of the AgNPs, as a blue shift is associated with a reduction in nanoparticle size (Haiss et al. 2007). Moreover, the total absorption peaks and the breadth of the absorption peaks are indicative of the size distribution and shape of AgNPs in the solution (Teponno et al. 2006). The presence of a single SPR band further validates the spherical and round shape of the AgNPs (Raza et al. 2016).

### SEM analysis of AgNPs

**Fig. 3:**
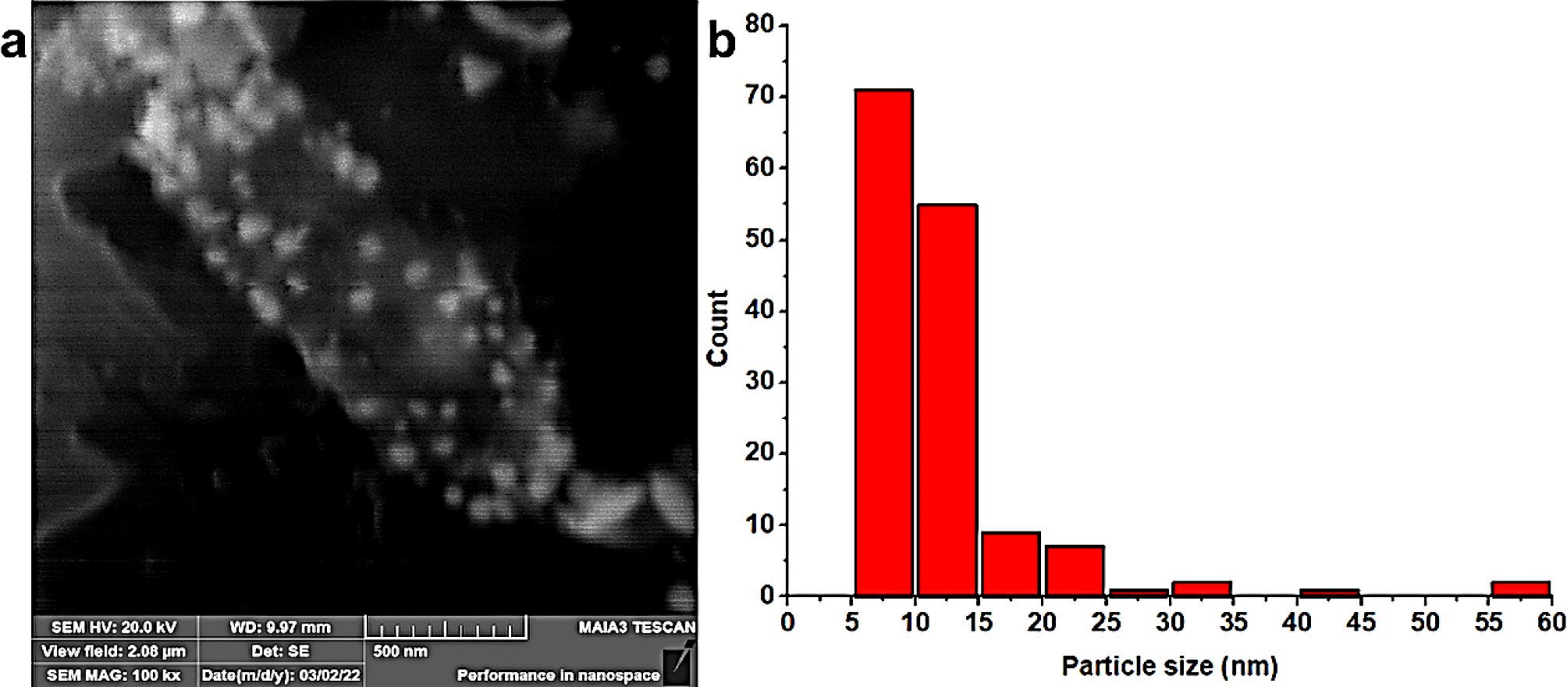
SEM micrograph of AgNPs synthesized from aqueous extract of *Viburnum grandiflorum* leaves (**a**) and particle size distribution histogram (**b**)

**Fig. 4:**
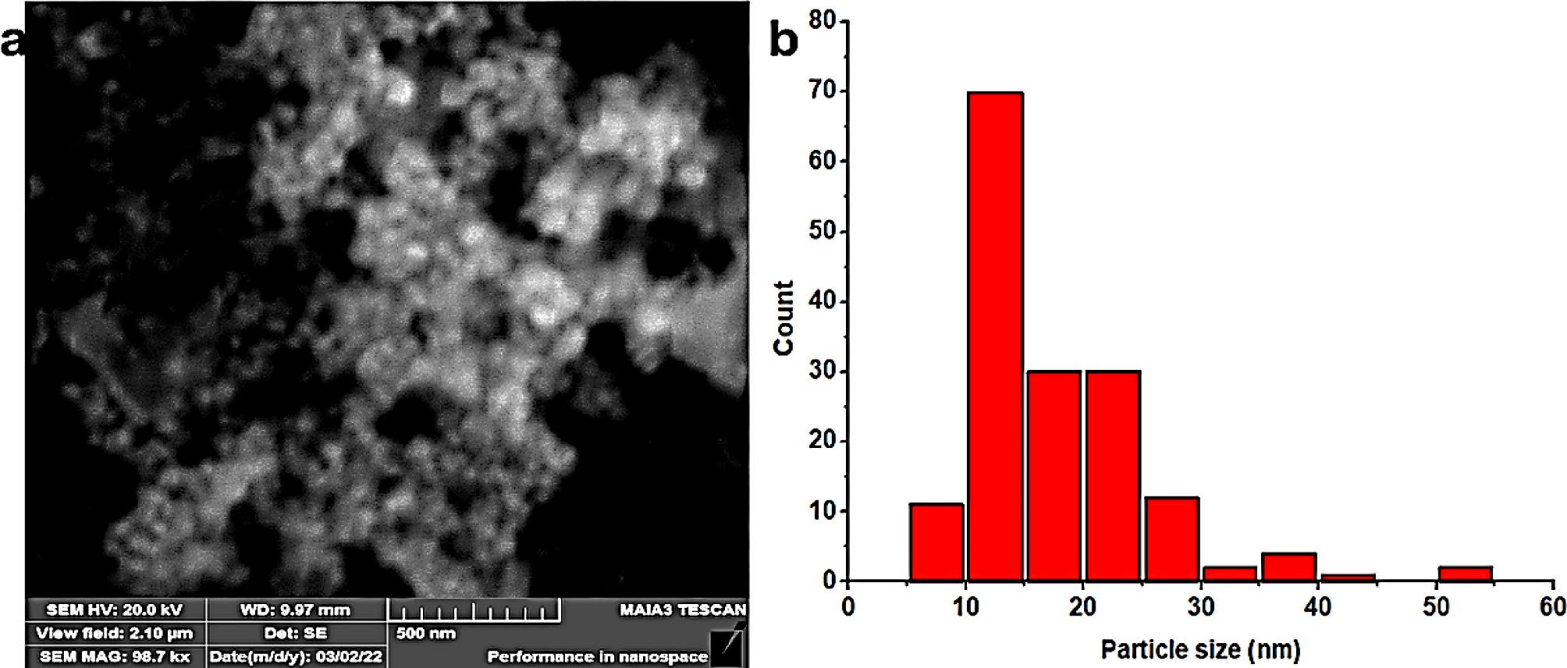
SEM micrograph of AgNPs synthesized from methanol extract of *Viburnum grandiflorum* leaves (**a**) and particle size distribution histogram (**b**)

The morphological characteristics of the synthesized AgNPs were examined using SEM. SEM image confirms that the AgNPs fabricated from aqueous extract exhibited a round shape with a mean size of 17.77 nm (Fig. 3). Similarly, the AgNPs synthesized from the methanol extract were also round, with a smaller mean size of 12.28 nm (Fig. 4). As depicted in Fig. 2, the SEM findings are aligned well with the results obtained from UV-Visible spectroscopy. Notably, the AgNPs synthesized from the methanol extract are smaller in size compared to those synthesized from the aqueous extract in consistent with prior studies (Huang et al. 2011; Yousaf et al. 2020).

### EDX analysis of AgNPs

**Fig. 5:**
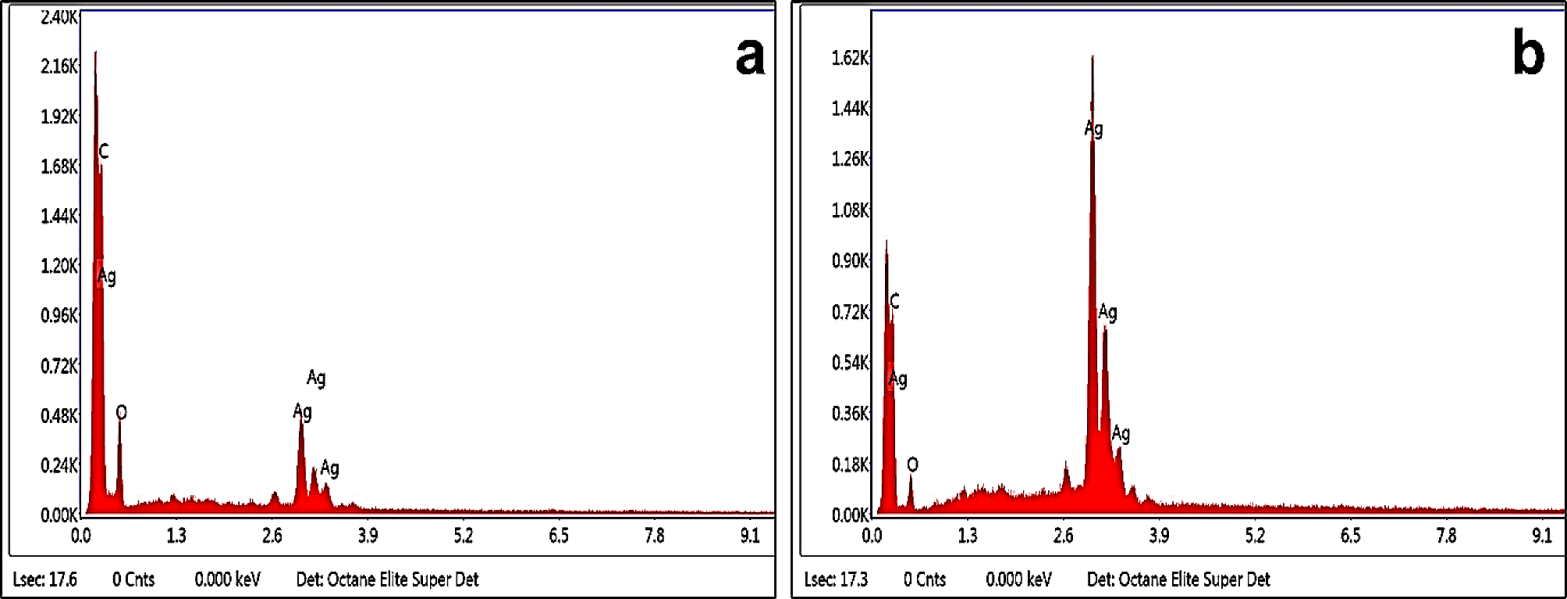
EDX spectra of AgNPs synthesized from aqueous (**a**) and methanol (**b**) extracts of *Viburnum grandiflorum* leaves

The elemental makeup of AgNPs was verified using an EDX detector integrated with FESEM. Strong silver (Ag) spectral peaks at about 3 keV were seen in the energy dispersive spectra as a result of metallic silver nano crystallites being absorbed by AgNPs’ SPR (Fig. 5). AgNPs have a distinctive optical absorption peak of about 3 keV because of SPR (Magudapatty et al. 2001). Notably, the signal is more pronounced in AgNPs fabricated from the methanol leaf extract of *V. grandiflorum*, indicating a higher purity of AgNPs in this case. These findings are consistent with a previous study on *Curcuma longa*-generated AgNPs, where a strong signal for Ag and a typical absorption peak at 3 keV were observed (Sathishkumar et al. 2010).

### X-ray diffraction

**Fig. 6:**
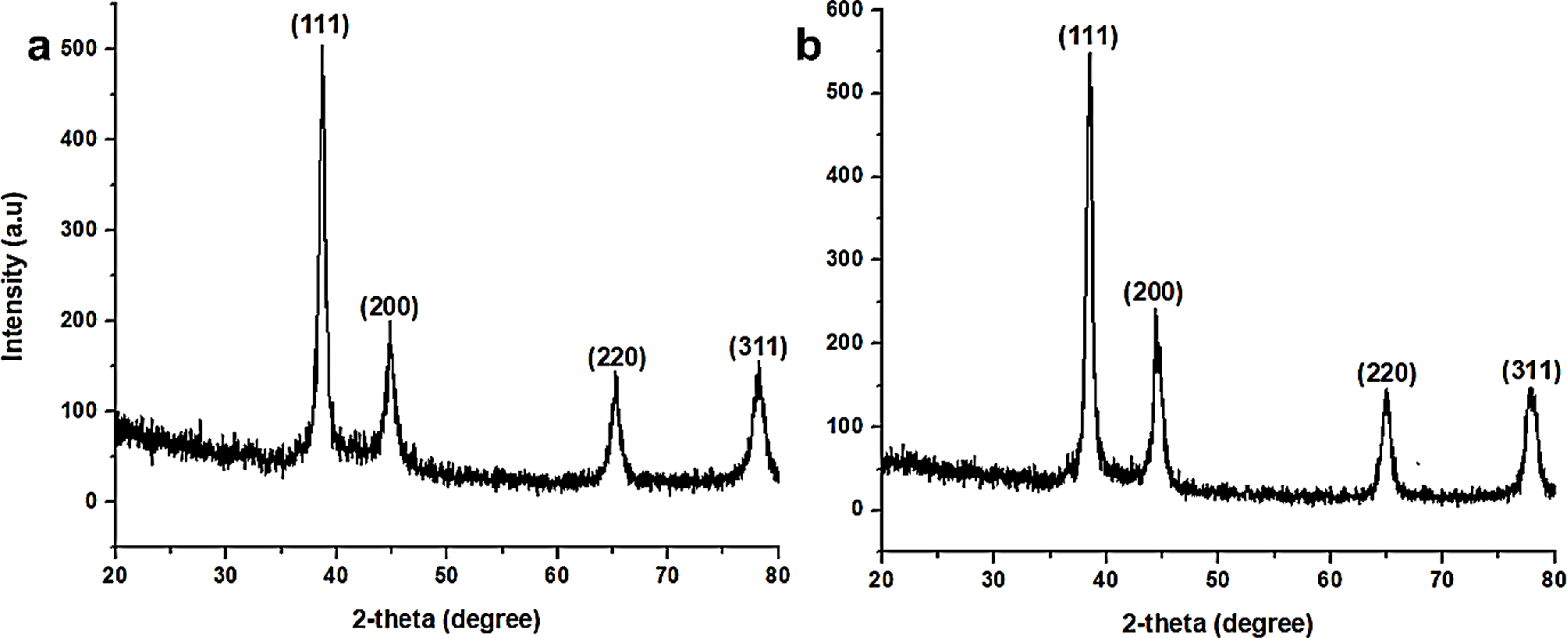
XRD patterns of AgNPs synthesized from aqueous (**a**) and methanol (**b**) extracts of *Viburnum grandiflorum* leaves

XRD study was applied to establish the crystalline phase and orientation of the bio-fabricated AgNPs. According to Fig. 6, the obtained diffraction peaks are at 38.95, 44.05, 65.00 and 79.45 for Aq-AgNPs and at 38.35, 45.45, 65.95, and 78.65 for met-AgNps, which relate to Miller indices of 111, 200, 220, and 311 planes (Prathna et al. 2011). The findings for the *V. grandiflorum* generated AgNPs were in good accord with JCPDS file no. 04-0783, and synthesized AgNPs were found to have face centered cubic symmetry. Although the size of the NPs is changed with the type of leaf extract, but the position of the diffraction peak remained unchanged (Mehmood et al. 2016). This shows that AgNPs were pure and very crystalline in composition, as evidenced by the strong and narrow diffraction peak in the XRD spectrum (Sarwer et al. 2022; Goyal et al. 2018).

### Antibacterial activity of AgNPs

**Fig. 7:**
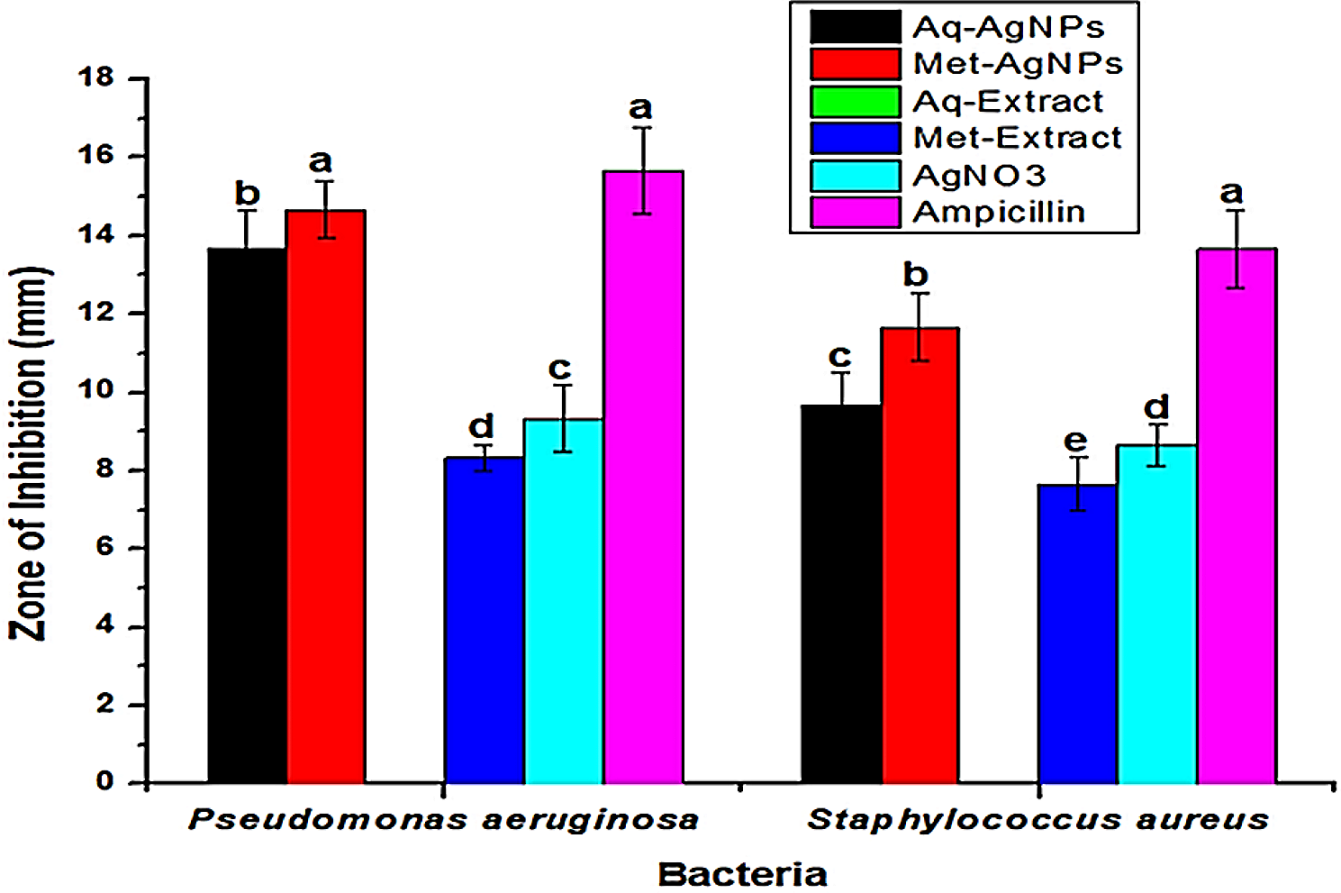
Antibacterial activity of AgNPs synthesized from aqueous and methanol extracts of *Viburnum grandiflorum* leaves

The antibacterial properties of AgNPs make them promising for treating bacterial infections (Haider and Kang 2015). In this study, the antibacterial application of AgNPs was investigated against *S. aureus* and *P. aeruginosa*. Unlike many studies that solely use the plant’s aqueous extract for AgNP synthesis, the use of different solvent extracts for AgNP synthesis is an innovative approach with potential advantages in terms of antibacterial activity. The results indicated that different solvent extracts influence the size of AgNPs, resulting in distinct antibacterial activities. Figure 7 presents the antibacterial results of AgNPs, demonstrating the high susceptibility of both *S. aureus* (Gram-positive) and *P. aeruginosa* (Gram-negative) bacterial strains to the AgNPs. Previous study also confirms the antibacterial activity of AgNPs against these bacteria (Sheng et al. 2022). The highest antibacterial activity was shown by Met-AgNPs against *P. aeruginosa* (14.66 ± 0.74 mm) as compared to Aq-AgNPs. This is attributed to the particle size, as Met-AgNPs were smaller in size than Aq-AgNPs. Small AgNPs may readily diffuse or may pierce bacterial cell membranes to stop the growth of the bacteria by interfering with the processes of their natural metabolism (Mubarak et al. 2018; Saravanan et al. 2015). Smaller AgNPs have more surface area than larger ones, which results in stronger antibacterial activity (Kvitek et al. 2008). Moreover, the stabilizing agent and functional groups present in the methanol extract may have been attached to the surface of the NPs and contributed to the higher activity of Met-AgNPs. Additionally, the polydispersity of synthesized nanoparticles and the plant extract originally used for nanoparticle synthesis may be strongly correlated with the antibacterial activity of biosynthesized nanoparticles. Interestingly, we observed that the Gram-negative bacterium (*P. aeruginosa*) was more susceptible to AgNPs than the Gram-positive bacterium (*S. aureus*). This difference may be attributed to the thin peptidoglycan layer in Gram-negative bacteria, along with an additional lipopolysaccharide outer membrane, facilitating the entry of released ions and NPs into the cell. Conversely, the thick peptidoglycan layer in the cell walls of Gram-positive bacteria, containing covalently linked teichoic and teichuronic acids, may act as a shield against the inhibitory effects of AgNPs (Shrivastava et al. 2007).

In the case of plant extracts, only the methanol extract exhibited antibacterial activity compared to AgNPs; the aqueous extract showed none. AgNPs, with their significantly larger surface area, smaller size, and surface-adhered biomolecules, likely contribute to their potent antibacterial activity (Ahmad et al. 2015). When AgNPs come into contact with bacterial surfaces, they can cause serious damage to the cell membrane and alter the structure of bacteria (Periasamy et al. 2012). Due to their small size, extensive surface area, and distinct electrostatic charge, AgNPs can efficiently interact with bacterial cells, easily accessing and influencing the cellular contents (Chen et al. 2010). Another important process likely involving in AgNPs’ antibacterial activity is that they are suspended in the solution. As a result, Ag ions are discharged, interact with sulfur-containing proteins in bacteria’s cell walls, and immediately change how those proteins function (Ovington 2004). Numerous studies have anticipated that the release of Ag ions by AgNPs is principally responsible for eliciting inhibitory effects against bacterial strains when interacting with the cell surface of microbes (Lee et al. 2005).

### Antioxidant activity of AgNPs

**Fig. 8:**
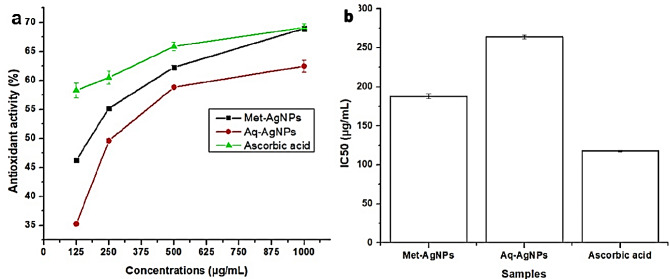
Antioxidant activity of AgNPs against DPPH (**a**) and IC_50_ value of AgNPs (**b**). The results are represented in means and standard error of means of 3 replicates. Different letters indicate the significant difference among the means calculated by ANOVA and LSD at *p* = 0.05

A DPPH scavenging assay was employed to evaluate the antioxidant activity of Aq-AgNPs, Met-AgNPs, and ascorbic acid. Figure 8 illustrates a significant difference in antioxidant activity, with a dose-dependent increase observed. For Aq-AgNPs, the recorded value increased from 35.29% at the lowest concentration (125 μg/mL) to 62.48% at the highest concentration (1000 μg/mL). In comparison, Met-AgNPs exhibited values of 46.27% and 68.96% for the respective concentrations, indicating a higher scavenging activity than Aq-AgNPs. The antioxidant results are also expressed in IC_50_ values (Fig. 8b), where Met-AgNPs demonstrated a lower IC_50_ value (188.00 ± 2.67 μg/mL) than Aq-AgNPs (264.01 ± 2.67 μg/mL). A lower IC_50_ value indicates higher antioxidant activity.

The presence of various functional groups on the surface of AgNPs may contribute to their elevated antioxidant activity. These findings suggest the potential use of AgNPs as an alternative antioxidant in the treatment of conditions induced by free radicals. Several studies have demonstrated that AgNPs produced from plant extracts exhibit high antioxidant activity (Sarwer et al. 2022; Khalil et al. 2012; Nagaich et al. 2016). The alkaloids, phenols, proteins, and other substances found in *V. grandiflorum* can donate hydrogen from their hydroxyl group (-OH) to free radicals, generating stable phenoxyl radicals (Chang et al. 2001).

### Anticancer activity of AgNPs

**Fig. 9:**
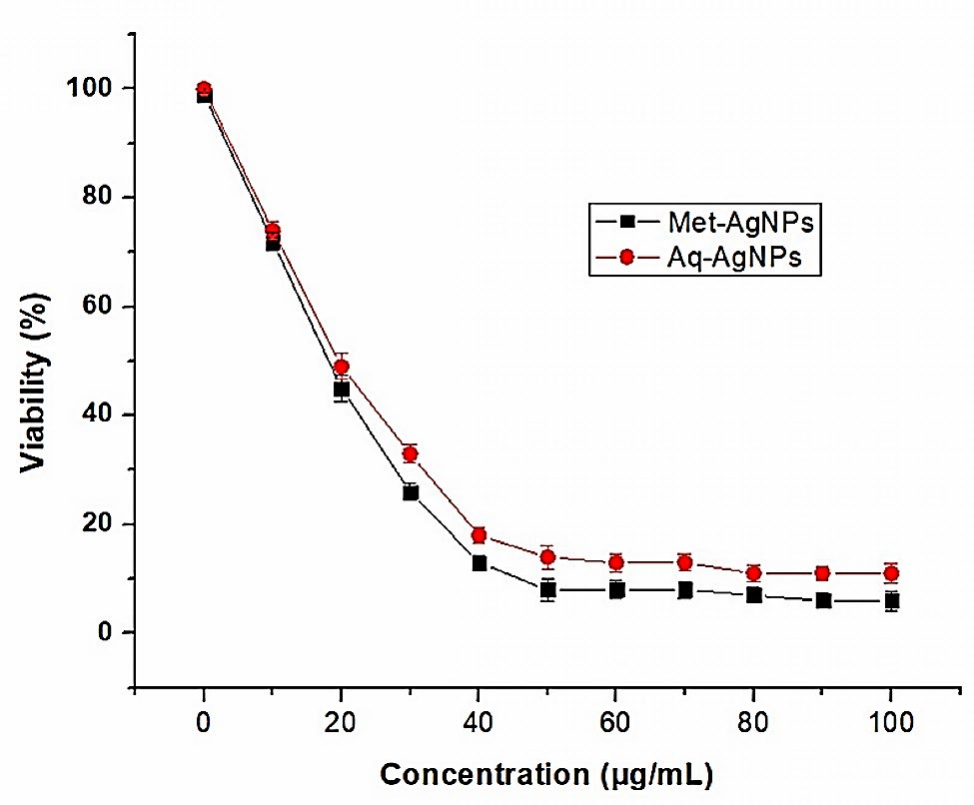
Cell viability of RD cell lines against AgNPs

*V. grandiflorum* generated AgNPs substantially reduced the cell viability of RD cell lines at a concentration of 10 to 100 μg/mL (Fig. 9). Aq-AgNPs decreased the RD cell lines’ viability from 100 ± 0.33 to 11 ± 1.67% when AgNPs concentration was raised from 0 to 100 μg/mL. Whereas, in the case of Met-AgNPs, the cell viability of RD cell lines was decreased from 100 ± 0.33 to 6 ± 1.78% when AgNP concentration was raised from 0 to 100 μg/mL, much lower than that of Aq-AgNPs. These results clearly show that Met-AgNPs, which are smaller in size than Aq-NPs, have more anticancer activity. Utilizing data from the MTT assay, the anticancer efficacy of AgNPs was quantified in terms of the half-maximal inhibitory concentration (IC50), as presented in Table 1.

The IC_50_ value of Aq-AgNPs and Met-AgNPs was 26.28 ± 1.44 and 21.49 ± 1.58 μg/mL, respectively. Again, IC_50_ values suggest the higher anticancer activity of Met-AgNPs. AgNPs’ enhanced cytotoxicity in RD cell lines may be due to their greater cell uptake and stability. AgNPs are not affected by p- glycoprotein efflux because of their smaller size and increased surface area, which permits them to enter cells via endocytosis (Gabizon 1992; Wei et al. 2009). Previous studies also suggest the great potential of biosynthesized AgNPs against cancer cell lines (Firdhouse and Lalitha 2013; Gajendran et al. 2014). Recent studies by AshaRani et al. (2009) and Nagajyothi et al. (2014) have demonstrated that AgNPs produced through green synthesis methods can inhibit the proliferation of human glioblastoma cells, A549 lung carcinoma, and MCF-7 breast cancer cells. The exact mechanism of action of AgNPs against cancer cells is not fully understood. Nevertheless, Xu et al. (2012) found that a silver NPs hydrogel induced DNA damage and the production of reactive oxygen species (ROS) in cancer cells, leading to cell death. In vitro studies have shown that AgNPs can also exhibit cytotoxic effects on normal cells. However, the extent of cytotoxicity depends on factors like particle size and concentration. Larger particles tend to be less toxic than smaller ones, and higher concentrations are more likely to cause harm. It’s important to emphasize that the toxicity of AgNPs is dose-dependent, and the concentrations used in scientific studies may not always reflect real-world exposure levels. Additionally, the findings can vary depending on the specific type of normal cells studied, as different cell types may respond differently to AgNPs (Akter et al. 2017).

**Table 1:** IC_50_ of AgNPs against RD cell lines

Cell lines	IC_50_ (μg/mL)	
	Met-AgNPs	Aq-AgNPs
RD cell line	21.49 ± 1.44	26.28 ± 1.58

It is noteworthy to consider the potential role of secondary metabolites in contributing to the antibacterial, antioxidant and anticancer potential of *Viburnum grandiflorum* derived green-fabricated silver nanoparticles. *V. grandiflorum* is known to contain a diverse array of secondary metabolites including flavonoids, phenolic acids, tannins and terpenoids as reported in previous literature (Suleman et al. 2018). These bioactive compounds are recognized for their inherent biological properties and have been linked to various health-promoting effects. Chen et al. (2021) describes 185 new and 228 known secondary metabolites from *Viburnum* genus between 2008 and 2020. Future investigations could explore the specific phytochemical constituents responsible for the observed antibacterial, antioxidant and anticancer activities. Incorporating such an analysis would provide valuable insights into the mechanistic basis of the bioactivity exhibited by the green-synthesized silver nanoparticles and further contribute to the understanding of *V. grandiflorum* medicinal potential.

## Conclusion

In this study, we successfully synthesized AgNPs using aqueous and methanol extracts of *V. grandiflorum*, employing an ecofriendly, cheap, and non-toxic method. The AgNPs synthesized from methanol extract were smaller in size compared to those from the aqueous extract. Both Aq-AgNPs and Met-AgNPs exhibited a spherical shape with a crystalline nature. These biosynthesized AgNPs demonstrated potent antibacterial activity against *P. aeruginosa* and *S. aureus* bacteria. Additionally, they showed antioxidant potential against DPPH. Furthermore, AgNPs effectively reduced the viability of RD cell line, with the methanol extract yielding more effective AgNPs compared to the aqueous extract. Overall, this study suggests that AgNPs generated by *V. grandiflorum* possess significant antibacterial, antioxidant, and anticancer activities, making them potentially valuable in pharmaceutical applications.

## Data Availability

All data associated with the study is presented in manuscript.
